# Urolithin A increases the natural killer activity of PBMCs in patients with prostate cancer

**DOI:** 10.3389/fphar.2024.1503317

**Published:** 2025-01-09

**Authors:** Vladimir Rogovskii, Vladimir V. Murugin, Nikolay Vorobyev, Sergey Popov, Nikolay Sturov, Alexey Krasheninnikov, Alexander Morozov, Marina Prokhorova

**Affiliations:** ^1^ Department of Molecular Pharmacology and Radiobiology, Pirogov Russian National Research Medical University, Moscow, Russia; ^2^ Department of Neuroimmunology, Federal Center of Brain Research and Neurotechnology of the Federal Medical-Biological Agency of Russia, Moscow, Russia; ^3^ Laboratory of Clinical Immunology, National Research Center—Institute of Immunology Federal Medical-Biological Agency of Russia, Moscow, Russia; ^4^ P. Hertsen Moscow Oncology Research Institute, The branch of the FSBI “National Medical Research Radiological Centre” (NMRRC) of the Ministry of Health of the Russian Federation, Moscow, Russia; ^5^ Department of Oncology, Radiotherapy and Reconstructive Surgery of I.M. Sechenov First Moscow State Medical University (Sechenov University), Moscow, Russia; ^6^ Department of General Practice, Medical Institute, Peoples’ Friendship University of Russia Named After Patrice Lumumba (RUDN University), Moscow, Russia; ^7^ Institute for Personalized Oncology, Center for Digital Biodesign and Personalized Healthcare, First Moscow State Medical University of the Ministry of Health of Russia (Sechenov University), Moscow, Russia

**Keywords:** urolithin A, natural killer activity, PBMCs, prostate cancer, computational prediction

## Abstract

**Background:**

The natural killer (NK) activity of peripheral blood mononuclear cells (PBMCs) is a crucial defense against the onset and spread of cancer. Studies have shown that patients with reduced NK activity are more susceptible to cancer, and NK activity tends to decrease due to cancer-induced immune suppression. Enhancing the natural cytotoxicity of PBMCs remains a significant task in cancer research.

**Methods:**

This study investigates the potential of urolithin A, a polyphenolic metabolite produced by the gut microbiota, to enhance the natural cytotoxicity of PBMCs in prostate cancer patients and healthy subjects. We investigated the possible role of aryl hydrocarbon receptor (AhR) in this capability of urolithin A. We analyzed the ability of PBMCs preincubated with urolithin A, AhR agonist or antagonist to kill K562 (human chronic myelogenous leukemia) target cells.

**Results:**

Our results demonstrate that urolithin A enhances the natural cytotoxicity of PBMCs in a dose-dependent manner. Specifically, at a concentration of 10 μM, urolithin A increased the NK activity of PBMCs from prostate cancer patients by an average of 23% (95% CI, 7%–38%). In addition, urolithin A modulates the level of cytokine production by PBMCs, decreasing the level of fractalkine, IL-8, and MCP-3. An AhR antagonist (CH223191, 1 μM) also increased NK activity, while an AhR agonist (β-naphthoflavone, 10 μM) did not increase NK activity and partially inhibited the urolithin A-induced enhancement.

**Conclusion:**

Urolithin A increases the NK activity of PBMCs from patients with prostate cancer and healthy subjects, and the AhR may be involved in this capability of urolithin A.

## 1 Introduction

The natural cytotoxicity of peripheral blood mononuclear cells (PBMCs) is one of the most important barriers to the onset and spreading of cancer. The natural cytotoxicity of PBMCs, also called a natural killer activity, is the ability of PBMCs, especially natural killer cells (NK cells), to quickly attack target cells (for instance, tumor cells) without the necessity of prior sensitization (Ran et al., 2022). Cytotoxicity of PBMCs is depleted in cancer patients (Janjic et al., 2022) and tumor entry is related to rapid functional impairment of NK cells (Dean et al., 2024).

Among different cancer types prostate cancer is one of the most common cancers worldwide, being the second most common cancer among men (Bergengren et al., 2023). In a recent study men with a low NK activity had five times higher odds of prostate cancer at biopsy (Vidal et al., 2019). And, it is of interest, that according to another recent study, NK cell infiltration in prostate cancers predicts improved patient outcomes (Zorko et al., 2024). However, not only NK cells are responsible for the natural cytotoxic activity of PBMCs. According to several studies, all major subsets of PBMCs can mediate innate cytotoxicity (Sturlan et al., 2009; Janjic et al., 2022). As a result, it is the NK activity of the entire PBMC population that contributes to antitumor immunity.

Thus, an important question arises, if there are ways to enhance the cytotoxic activity of PBMCs against cancer cells? Urolithin A, a polyphenol metabolite produced by gut microbiota, shows promise for cancer therapy. It has a favorable safety profile and exhibits immune-modulatory and anticancer properties, making it a potential therapeutic for cancer treatment and prevention (Rogovskii, 2022b; D'Amico et al., 2021; Ginefra et al., 2024). Urolithin A acts through various mechanisms, notably by modulating the aryl hydrocarbon receptor (AhR). AhR, originally studied as an environmental sensor for toxins and dietary ligands, regulates metabolic responses and increases cytochrome P450 enzymes (CYP) for xenobiotic metabolism (Riaz et al., 2022). In addition to external ligands, AhR also interacts with endogenous ligands to regulate multiple systems, particularly immunity.

Data indicate that urolithin A acts as an AhR antagonist (Rogovskii, 2022b; Muku et al., 2018), though some consider it a partial agonist (Shen et al., 2021). This is not contradictory, as different ligands can produce varied effects on AhR, with some agonists interfering with others (Denison and Faber, 2017). The effects of urolithins on PBMCs natural cytotoxicity remained unexplored. In this study, we examined urolithin A’s ability to enhance the cytotoxicity of PBMCs from prostate cancer patients against cancer cells and assessed the role of AhR in this effect.

## 2 Materials and methods

### 2.1 Patients and donors

We analyzed the ability of PBMCs preincubated with urolithin A, AhR agonist or antagonist to kill K562 (human chronic myelogenous leukemia, ATCC) target cells. K562 cells are widely used as a target cell to assess the cytotoxicity of PBMCs because these cells lack major histocompatibility complex (MHC) molecules resulting in the induction of NK activity (Tremblay-McLean et al., 2019). Conformity of the used cell line to the ATCC reference database was confirmed by the short tandem repeat (STR) analysis using the COrDIS Plus test system (GORDIZ, Russia).

Peripheral blood samples (8–10 mL of whole blood, heparin tubes) were obtained from patients with prostate adenocarcinomas (n = 20). Their mean age was 66 years (range, 55–77 years). Controls (n = 5) included peripheral blood of healthy, tumor-free, male individuals. Their mean age was 59 years (range, 54–76 years). All subjects gave informed consent to the study. The study was performed in line with the principles of the Declaration of Helsinki. Approval was granted by the Ethics Committee of Pirogov Russian National Research Medical University. Patients with human immunodeficiency virus (HIV)/hepatitis C virus/hepatitis B virus infection, diabetes, chronic inflammatory conditions, treated with chemotherapy or radiotherapy, or subjected to immunosuppressive therapies, were excluded from the study. Clinical features of the patients with prostate cancer are shown in Table 1.

**TABLE 1:** Clinical features of the patients with prostate cancer and healthy subjects.

Patients with prostate cancer				
ID	Age	TNM	PSA (ng/mL)	Gleason score
1	69	pT2N0M0	14.00	3 + 3
2	62	pT2N0M0	6.90	3 + 3
3	64	ypT2N0M0	5.49	3 + 3
4	64	pT2N0M0	3.68	3 + 3
5	58	pT3bN0M0	35.30	3 + 3
6	77	pT3aN0M0	9.75	3 + 4
7	75	pT3aN0M0	6.50	3 + 3
8	68	pT2N0M0	6.37	4 + 4
9	67	ypT3aN1M0	22.96	4 + 5
10	64	pT2N0M0	5.40	3 + 4
11	65	pT2N0M0	17.00	4 + 4
12	68	pT2N0M0	9.00	3 + 4
13	60	pT2N0M0	4.55	3 + 4
14	55	pT3aN1M0	6.00	4 + 3
15	67	ypT2N0M0	33.00	3 + 4
16	63	pT2N0M0	5.00	3 + 4
17	67	pT2N0M0	2.50	3 + 4
18	69	pT2N0M0	2.40	4 + 3
19	61	pT3aN1M0	38.24	3 + 3
20	68	ypT2N0M0	16.00	3 + 3
Healthy subjects				
1	76	N/A	2.4	N/A
2	53	N/A	0.6	N/A
3	54	N/A	1.1	N/A
4	54	N/A	0.8	N/A
5	56	N/A	1.4	N/A

TNM – classification of malignant tumors. T (tumor) - describes the size of the primary tumor and the invasion into adjacent tissues; N (nodes) - describes regional lymph node involvement of the tumor; M (metastasis) - describes the presence of distant metastases of the primary tumor (Rosen and Sapra, 2024).

PSA – the level of prostate-specific antigen in the blood.

Gleason score – the primary histological assessment tool used to grade prostate malignancies. The lower the Gleason score, the more similar the cancer cells look to normal cells. A Gleason score of 1 would appear almost normal, while a Gleason score of 5 would have no glandular characteristics at all. The two numbers represent the Gleason grade of the predominant pattern and the grade of the next most common pattern (Munjal and Leslie, 2024).

N/A, not applicable.

### 2.2 Study of NK activity of PBMCs cultured in the presence of studied substances

The NK activity of PBMCs (we use cytotoxicity of PBMCs as the synonym of NK activity of PBMCs) was studied by flow cytometry by killing K562 target cells labeled with CFSE (5-, 6-carboxyfluorescein diacetate-succinimidyl ester) according to the method described in detail in (Murugin et al., 2011). PBMCs were isolated from venous blood by using Ficoll density gradient centrifugation, washed three times, and resuspended in complete culture medium RPMI containing glutamine (2 mmol/L, Paneco, Russia), and 10% fetal calf serum (Biosera, South Africa).

Freshly isolated PBMCs (10^6^/1 mL) were mixed with studied substances (urolithin A (6762, Tocris Bioscience, United Kingdom), CH223191 (antagonist of AhR, C8124, Sigma-Aldrich, United States) and β-naphthoflavone (agonist of AhR, A18543, Alfa Aesar, United States) in complete culture medium at various concentrations in 15-mL polypropylene tubes and cultured for 24 h (37°C; 5% CO2). PBMCs in a complete culture medium were used as a negative control; PBMCs in a complete culture medium and IFN-α (Invitrogen, United Kingdom) at a concentration of 1,000 IU/mL were used as a positive control for stimulation of the NK activity [as described in the article of Karsonova and coauthors (Karsonova et al., 2013)]. After the end of incubation, some supernatants were collected for multiplex plasma cytokine analysis, then PBMCs were washed once in RPMI and resuspended in a complete culture medium to test for NK activity. Some of the cells were used to make lysates for further PCR.

The NK activity of the PBMCs was measured by using a flow cytometry-based assay. Briefly, K562 target cells were labeled with CFSE (Invitrogen, Paisley, United Kingdom) and plated in 96-well U-bottom plates at 8 × 10^4^/well. Effector PBMCs were added at 4 × 10^5^/well such that the E:T ratio was 50:1 (in duplicates). In previous work different E:T ratios were tested ranging from 3.125:1 to 50:1 (Murugin et al., 2011). Significant and reliable differences between the groups (donors and patients) were observed at E:T ratio of 50:1 (Murugin et al., 2011), therefore, this ratio was used in the current work. To control for spontaneous target cell death, an additional two wells containing only target cells were prepared. The cells were incubated for 4 h (37°C; 5% CO2) and then resuspended and stained for 10 min with propidium iodide (PI, Sigma-Aldrich, United States) at 1 μg/mL. Duplicates were pooled, and the cells were analyzed by using a flow cytometer (FacsCalibur with CellQuest software, Becton Dickinson). In each sample, the percentage of dead targets was determined as the percentage of PI + events among CFSE + events. Specific killing was calculated as follows: % specific killing = [(% experimental target death − % spontaneous target death)/(100 − the % spontaneous target death)] × 100.

### 2.3 RNA extraction and quantitative real-time PCR (qPCR)

Total RNA was extracted using HiPure Total RNA Kit (Magen Biotechnology, China) according to the manufacturer’s specifications. The synthesis of cDNA for qPCR was carried out using the RevertAid RT Reverse Transcription Kit (Thermo Scientific, United States). qPCR for CYP-1A and CYP-1B in PBMCs was carried out using the real-time PCR system (Roche, Lightcycler 96) with the reagent mix containing SYBR Green (Evrogen, Russia).

For the qPCR experiments, primers against the sequences of human CYP-1A1 (5′-GAT​TGA​GCA​CTG​TCA​GGA​GAA​GC-3′, 5′-CCA​AAG​AGG​TCC​AAG​ACG​ATG​TTA-3′), CYP-1B1 (5′-CTC​AAC​CGC​AAC​TTC​AGC​AAC​TTC-3′, 5′- AGA​GAG​GAT​AAA​GGC​GTC​CAT​CAT-3′), and the housekeeping gene glyceraldehyde 3-phosphate dehydrogenase (GAPDH) (5′-TGC​ACC​ACC​AAC​TGC​TTA​GC-3′, 5′-GGC​ATG​GAC​TGT​GGT​CAT​GAG-3′) (Vorontsova et al., 2023) were purchased from Syntol (Moscow, Russia). The cycling conditions were 95°C for 300 s followed by 40 cycles of denaturing at 95°C for 15 s and annealing/extension for 45 s at 60°C. Reactions were carried out in duplicate. A melt curve analysis was performed to confirm the specificity of qPCR products. Relative gene expression was calculated with the 2^−ΔΔCT^ method. Results were normalized to the expression levels of the untreated controls, represented as the black columns.

### 2.4 Multiplex plasma cytokine analysis

The level of various immunoregulatory factors in the cell culture of PBMCs was measured using a commercial kit (MILLIPLEX MAP Human Cytokine/Chemokine Magnetic Bead Panel—Premixed 38 Plex, HCYTMAG-60K-PX38, Millipore Sigma, United States) using the MAGPIX (Luminex Corp, United States). The detected cytokines included sCD40L, EGF, Eotaxin/CCL11, FGF-2, Flt-3 ligand, Fractalkine, G-CSF, GM-CSF, GRO, IFN-α2, IFN-γ, IL-1α, IL-1β, IL-1ra, IL-2, IL-3, IL-4, IL-5, IL-6, IL-7, IL-8, IL-9, IL-10, IL-12 (p40), IL-12 (p70), IL-13, IL-15, IL-17A, IP-10, MCP-1, MCP-3, MDC (CCL22), MIP-1α, MIP-1β, TGF-α, TNF-α, TNF-β, VEGF. The analysis was performed according to the manufacturer’s instructions. xPONENT 4.1 Software was used to obtain the raw data. The concentrations of 38 cytokines were determined using standard curves.

### 2.5 Statistical analysis

Statistical analysis of the data was performed using Prism 8 software. Shapiro-Wilk test and QQ plots were used to evaluate the distribution of the variables. In the analysis of NK activity and in the analysis of PCR data t-test was used for comparison with control group, and ANOVA followed by two-stage step-up method of Benjamini, Krieger and Yekutieli was used for multiple comparisons. Multiple t-test followed by two-stage step-up method of Benjamini, Krieger and Yekutieli was used for analysis of multiplex cytokine assay data.

### 2.6 Computational prediction of AhR modulation by urolithin A

We have used machine learning to perform computational prediction of AhR modulation by urolithin A. The source of data for the training set was the AICD database [an integrated “Anti-Inflammatory Compounds Database” (AICD) (Wang et al., 2019)], from which we have used data on half-maximal concentrations (EC50) required to activate the AhR. We have found molecular structure in SMILES format for 212 compounds. Python was used to create the model. After extracting the names of substances for which EC50 for the AhR was presented from the original database, the structures of the substances were obtained in MOL format using the RDKit library. Next, based on the MOL format, molecular fingerprints ECFP (Extended-Connectivity Fingerprints) were created for each substance, which makes it possible to present detailed data on the features of the molecular structure in a convenient form for machine learning (bit string). The radius for obtaining data for each atom was 4, and the size of the fingerprint line was 2048 bits.

Cause of the relatively small sample size, we have used it for building the classification model–to find whether the studied substance might strongly activate the AhR or weakly activate the AhR. In our database, the median EC50 value was 1,748 nM. In this regard, we divided all substances into those that strongly activate the AhR, with an EC50 of less than 1,748 nM, and those that weakly activate this receptor, with an EC50 of more than 1,748 nM.

**FIGURE 1:**
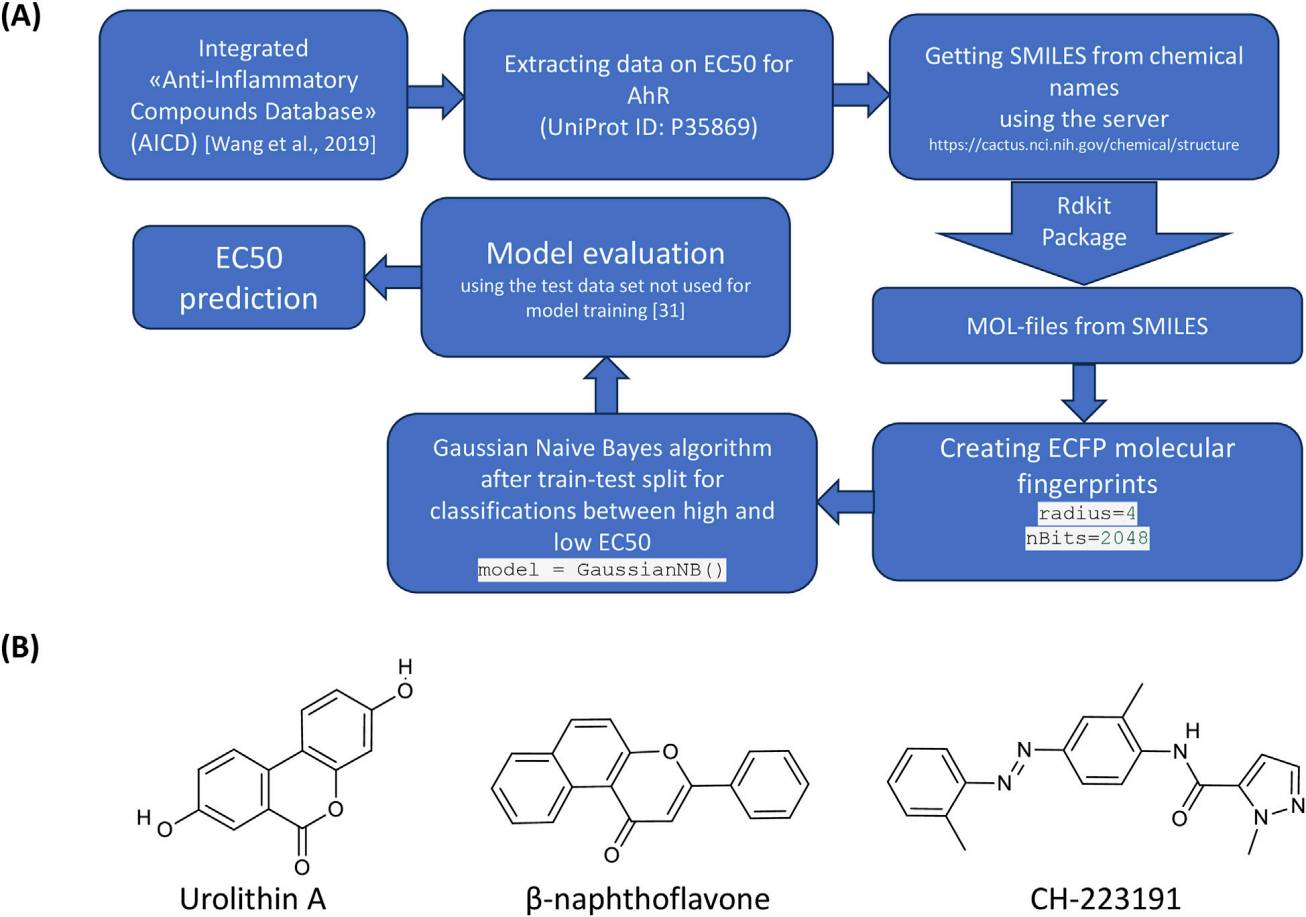
Analysis of the structures of urolithin A, AhR agonist and antagonist. **(A)** The workflow of data mining. **(B)** Chemical structures of urolithin A, AhR agonist (β-naphthoflavone) and antagonist (CH-223191).

A Naive Bayes classifier was used to find the relationship between molecular structure and EC50. To train and test the algorithm, a division into a training and test set was used (test set of the data that was not used when training the model, the size of the test set was of 20%). The workflow of computational prediction is presented in Figure 1A. The chemical structures of urolithin A, AhR agonist (β-naphthoflavone) and antagonist (CH-223191) are shown in the Figure 1B.

## 3 Results

### 3.1 Urolithin A increased the NK activity of the PBMCs in patients with prostate cancer and healthy subjects

**FIGURE 2:**
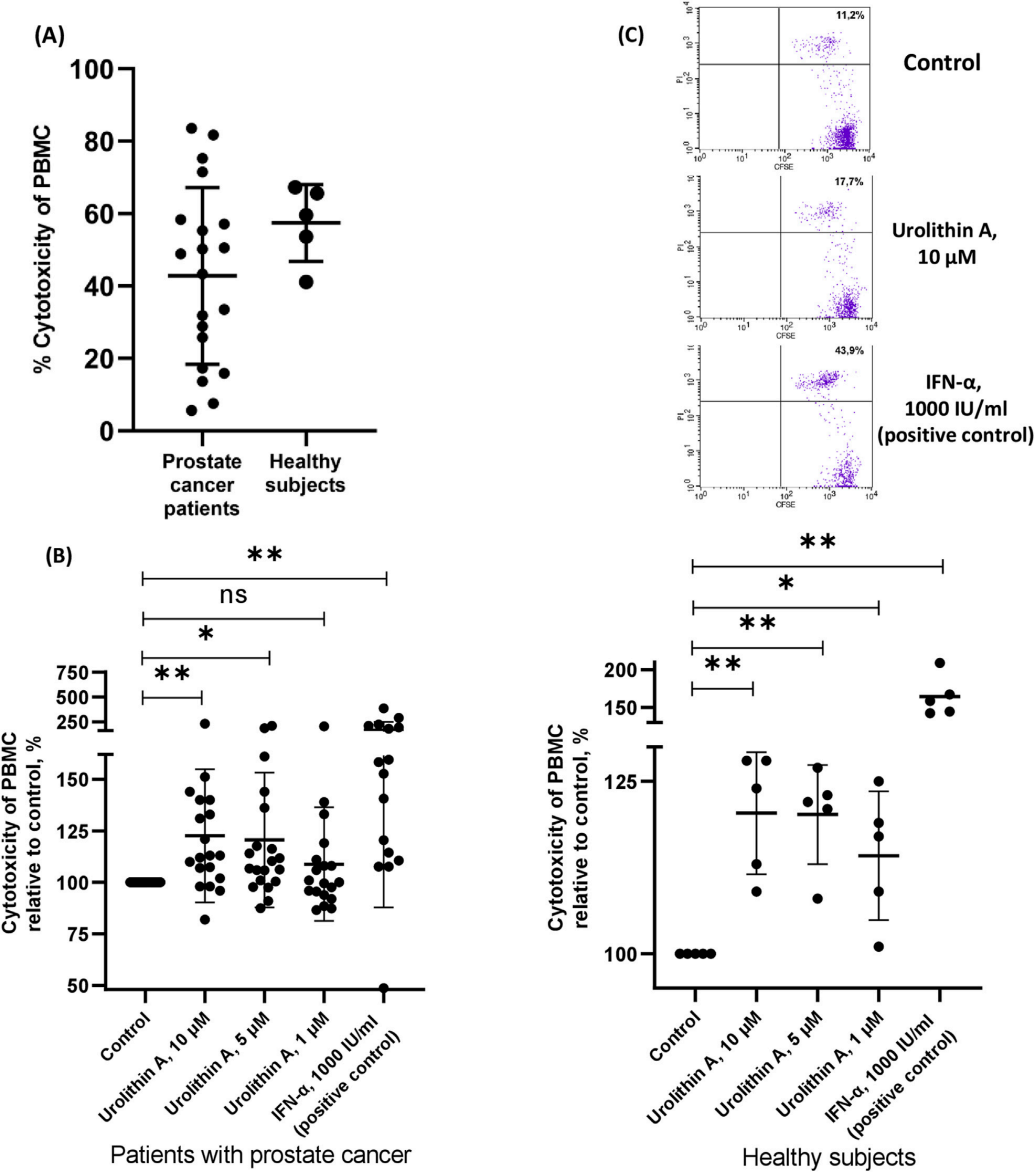
Urolithin A increased the NK activity of the PBMCs. **(A)** Cytotoxicity of PBMCs from patients with prostate cancer and healthy subjects (control samples). **(B)** The influence of urolithin A on cytotoxicity of PBMCs from patients with prostate cancer and healthy subjects. Data are expressed relative to the cytotoxicity of PBMCs in control samples, which are taken as 100%. Each dot corresponds to one sample and lines represent mean ± SD. *p < 0.05; **p < 0.01. **(C)** Representative flow-cytometric analysis of CFSE and PI labeled target cells (K562) in the coculture experiment with PBMCs from prostate cancer patient. Dead target cells (CFSE + PI+) are visualized in the upper right quadrant. The percentage of dead target cells is indicated.

We evaluated the NK activity of the PBMCs against target K562 cells in prostate cancer patients and healthy subjects (Figure 2A). We found that the addition of urolithin A increased the cytotoxic ability of PBMCs from patients with prostate cancer and healthy subjects (Figures 2B, C). For instance, urolithin A (10 μM) caused the mean increase of the NK activity of PBMCs by 23% (95% CI, 7%–38%). A lower concentration of urolithin A (5 μM) caused an average increase of the NK activity of PBMCs by 21% (95% CI, 5%–36%).

### 3.2 The role of AhR in the urolithin A-dependent increase of NK activity of PBMCs

**FIGURE 3:**
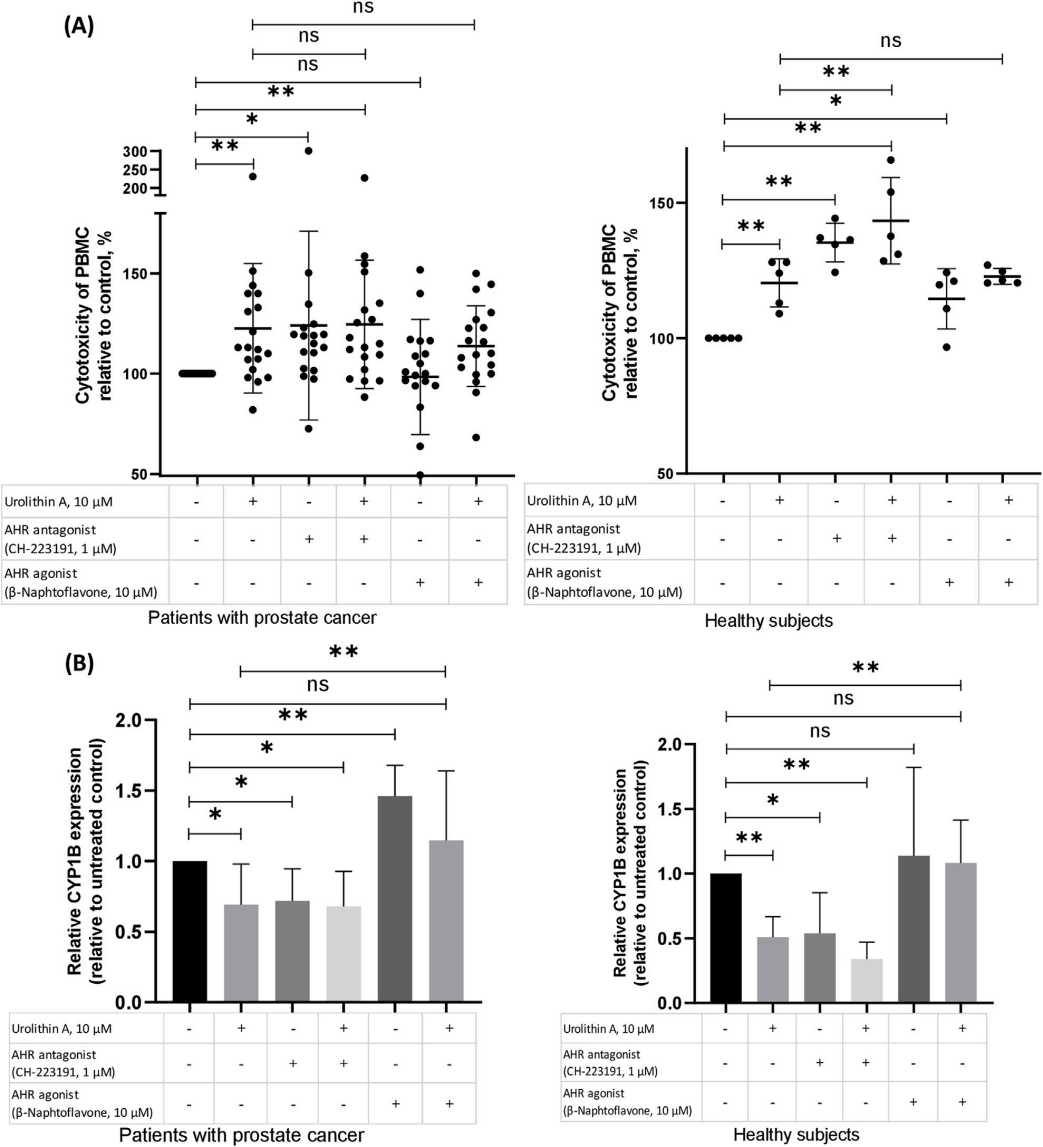
The role of AhR in the urolithin A-dependent increase of NK activity of PBMCs. **(A)** The role of the AhR in the urolithin A-mediated increase of natural cytotoxicity of PBMCs in patients with prostate cancer and healthy subjects. PMBCs (10^6^ per 1 mL), obtained from prostate cancer patients or healthy subjects, were preincubated for 1 h with an antagonist of the AhR (CH-223191, 1 μM) or agonist of the AhR (β-naphtoflavone, 10 μM), whereafter urolithin A (10 μM) was added to the cultures. After 24 h, the cells were washed and used for evaluation of natural cytotoxic ability according to the aforementioned procedure (see the *Methods* section). Data are expressed relative to the cytotoxicity of PBMCs in control samples, which are taken as 100%. Each dot corresponds to one sample and lines represent mean ± SD. *p < 0.05; **p < 0.01. **(B)** Effects of urolithin A on the AhR-regulated expression of CYP1B in patients with prostate cancer (N = 7) and healthy subjects (N = 5). PMBCs (10^6^ per 1 mL), obtained from prostate cancer patients or healthy subjects, were pre-incubated for 1 h with an antagonist of the AhR (CH-223191, 1 μM) or agonist of the AhR (β-naphtoflavone, 10 μM), whereafter urolithin A (10 μM) was added to the cultures for 24 h incubation. Real-time PCR analysis was performed as described in the methods section. Changes in mRNA expression were normalized to CABDH. The boxes in the graphs correspond to the means and the whiskers indicate the SD. *p < 0.05; **p < 0.01.

We have studied whether AhR is involved in the capability of urolithin A to induce NK activity of PBMCs (Figure 3). For this purpose, we have studied the influence of urolithin A on the NK activity of PBMCs in the presence of an agonist (β-naphtoflavone) or antagonist (CH-223191) of AhR (Figure 3A).

In our experiments, the antagonist of AhR, similar to urolithin A, increased the NK activity of PBMCs. In contrast, the agonist of AhR hasn’t shown such influence on the NK activity of PBMCs from patients with prostate cancer. Moreover, the agonist of AhR reduced to some extent urolithin A-mediated increase in PBMCs NK activity in patients with prostate cancer (p = 0.3).

Next, for further studying of the role of AhR in the urolithin A-dependent increase of PBMCs NK activity, we have examined the gene expression of target enzymes of AhR – CYP1A and CYP1B. Urolithin A suppressed the expression of CYP1B in both groups. Likewise, the antagonist of AhR suppressed the expression of CYP1B. Agonist of AhR increased CYP1B expression, mostly in PBMCs from patients with prostate cancer. And, finally, the agonist of AhR reduced the urolithin A-mediated decrease of CYP1B in both groups (Figure 3B).

### 3.3 Computational prediction of AhR modulation by urolithin A

We have made a structure-activity classification model, which allows us to predict the high activating potential (agonism) or low activating potential of the studied compounds to the AhR. When tested on the test set, the F1 Score was 0.727 (the harmonic mean between precision and recall). The accuracy of the model (AUC—Area Under the Curve) was 0.733.

Surprisingly, using this model, we have found that urolithin A belongs to the group of substances with relatively high potential to activate the AhR (EC50 less than 1,748 nM), as well as beta-naphthoflavone. Based on our model, the compound CH223191 (the antagonist of AhR), belongs to the group of substances with relatively low potential to activate the AhR, which suits to its role as an antagonist of AhR receptor. We have no dataset containing sufficient numbers of IC50 for AhR, that’s why only the EC50 dataset was used.

### 3.4 Effects of urolithin A, AhR agonist and antagonist, and their combinations on the cytokine production by PBMCs of patients with prostate cancer and healthy subjects

**FIGURE 4:**
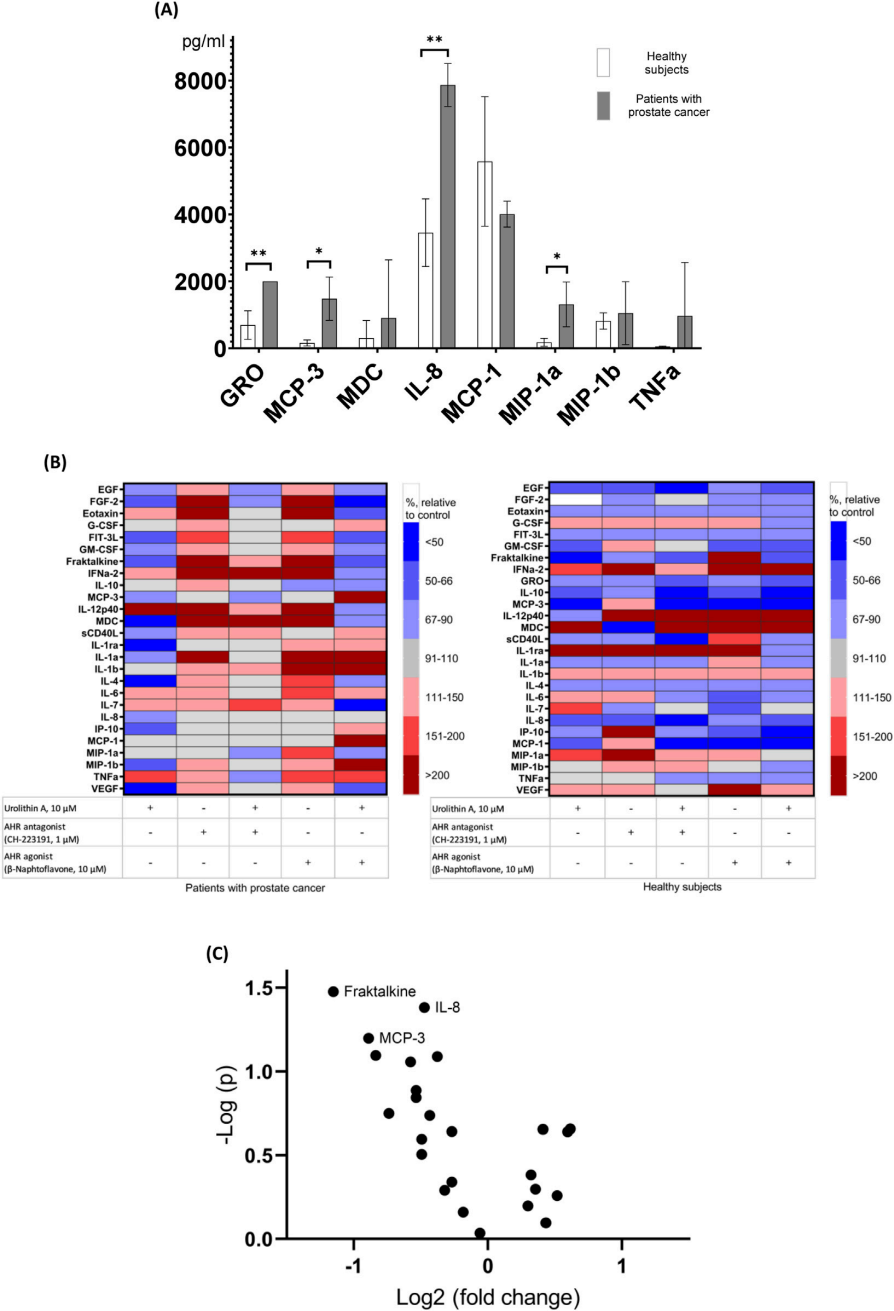
Effects of urolithin A, AhR agonist and antagonist, and their combinations on the cytokine production by PBMCs. **(A)** Comparison of absolute levels of cytokines produced by unstimulated PBMCs from patients with prostate cancer and healthy subjects (values above 1,000 pg/mL are shown). *p < 0.05; **p < 0.01. **(B)** Effects of urolithin A, AhR agonist and antagonist, and their combinations on cytokine production by PBMCs from patients with prostate cancer (N = 4) and healthy subjects (N = 3). Cluster heat map of analyte profiles (>10 pg/mL) in the studied groups of prostate cancer patients and healthy subjects. Each row in the figure represents an analyte (cytokines, chemokines, growth factors, etc.), each column represents a set of samples. Areas of red or blue indicate that the protein produced is increased or decreased, respectively, compared to the untreated control. The white area indicates that there was virtually no change in protein production compared to the untreated control. **(C)** The influence of urolithin A on the cytokine production by PBMCs of patients with prostate cancer and healthy subjects (volcano plot). Cytokines with levels above 10 pg/mL are shown.

Since, apparently, antagonism of the AhR is not the only mechanism of increase of NK activity by urolithin A, we have preliminarily studied the influence of urolithin A (also in the absence and in the presence of AhR agonist or antagonist) on the production of panel of inflammatory cytokines by PBMCs of patients with prostate cancer and healthy subjects (Figure 4). We have used multiplex cytokine assay to preliminarily identify possible targets of urolithin A among cytokines for further studies. Some of studied cytokines can be involved in the increase of NK activity (e.g., IFN-α) (Karsonova et al., 2013), while some of them can be involved in the decrease of NK activity (e.g., IL-6 and IL-8) (Wu et al., 2019). According to our data, unstimulated PBMCs from prostate cancer patients produced significantly higher levels of a number of immunoregulatory factors (GRO, MCP-3, MIP-1a, IL-8) than PBMCs from healthy subjects. On the one hand, urolithin A caused a decrease in the production of immunoregulatory factors, which was most noticeable for fractalkine, IL-8, and MCP-3. On the other hand, urolithin A increased IFN-α levels to some extent, but more experiments are needed to draw more reliable conclusions.

## 4 Discussion

Our data indicate that urolithin A increases the NK activity of PBMCs in patients with prostate cancer and healthy subjects. According to various studies, increasing the NK activity of immune cells is a promising strategy for immune-mediated therapy of cancer (Kim et al., 2019; Khanna et al., 2021).

Medium and high cytotoxic activity of PBMCs is associated with reduced cancer risk, whereas low activity is associated with increased cancer risk (Imai et al., 2000). For instance, the study of Sue In Choi has revealed that patients with low NK activity were more likely to have lung cancer (Choi et al., 2019). In line with this, NK activities of PBMCs from breast cancer patients were significantly lower as compared with those of healthy subjects (Dewan et al., 2009). Therefore, increasing NK activity is likely important in the prevention and treatment of various types of tumors. To be specific, in this work, we chose to study the NK activity of patients with one specific cancer—prostate cancer. In the future, it is important to conduct similar studies in other types of cancer (Kandarian et al., 2017).

The immune system is the main barrier to the onset of cancer. Increasing the immune capability to detect and kill cancer cells is an attractive strategy that was used over a hundred years ago (Coley’s vaccine) (Carlson et al., 2020). A modern extension of this approach is immunotherapy (for instance, checkpoint inhibitors and CAR T cells). Advances in immunotherapy have made a breakthrough in the treatment of cancer. However, in most cases, patients do not respond to immunotherapy and it has various side effects, especially severe autoimmune complications (Dobosz et al., 2022). The question is whether there could be a therapy that improves antitumor immunity while having a favorable safety profile. Such therapy is unlikely to provide rapid relief but could be suitable for long-term use as a disease-modifying therapy, which can be defined as a long-term treatment that has a beneficial outcome on the course of cancer (Rogovskii, 2022a).

Urolithin A has been shown to possess various anti-inflammatory, immune-modulatory and anticancer effects (Rogovskii, 2022b; Gimenez-Bastida et al., 2020). Of note, recently it was shown that urolithin-A promotes CD8^+^ T Cell-mediated cancer immunosurveillance via activation of FOXO1 (Ginefra et al., 2024). The concentrations of urolithin A we used (1–10 μM) are close to the concentrations of urolithins in blood and other biological fluids after treatment with urolithin A (or the source of parent substances of urolithin A such as pomegranate juice) (Muku et al., 2018; Garcia-Villalba et al., 2022). In the case of topical application, the concentration of urolithin A could be higher, opening up the prospect of urolithin A as a potential drug for skin cancer and precancerous conditions. Thus, the ability of urolithin A to increase the cytotoxicity of PBMCs might be one of the mechanisms of the anticancer action of urolithin A, which was shown in various *in vivo* models (Rogovskii, 2022b).

Our data have shown that antagonism of AhR might be among the mechanisms of the urolithin A anticancer effects. As mentioned above, AhR is a promising target in cancer therapy. In addition to exogen ligands, various endogenous ligands of AhR are involved in the regulation of a wide range of systems, especially immunity. AhR has numerous effects on T- and B-cells, macrophages, Th1/Th2 cell balance, and Th-17 cells/regulatory T cells balance, thus, playing a significant role in the regulation of immune tolerance in health and disease (Riaz et al., 2022). For instance, kynurenines, the products of IDO (indoleamine 2,3-dioxygenase)-mediated tryptophan metabolism, are ligands of AhR. It has been reported that in cancer activation of the kynurenine pathway has been associated with tumor progression, metastasis, and chemoresistance (Basson et al., 2023). Thus, the inhibiting of pathologic AhR activation is the promising method to reroute immunity toward tumor rejection (Basson et al., 2023; Hanieh et al., 2023).

In our experiments, an AhR antagonist mostly mimicked urolithin A’s effects on the NK activity, while an AhR agonist partially suppressed the urolithin A-induced increase in NK activity. At the level of CYP1B expression, which increases in response to AhR activation, we have seen similar effects. Urolithin A decreased CYP1B expression and the AhR antagonist showed the same effect as urolithin A. Adding the agonist of AhR reversed the effect of urolithin A.

Computer modeling of the structure-activity relationship showed that urolithin A might play a role as the agonist of AhR receptors. In some studies, urolithin A also appears as the partial agonist of AhR (Shen et al., 2021). It has been shown that different agonists of AhR can cause different effects (Shen et al., 2021). Thus, it might be assumed that urolithin A binds to AhR, preventing the action of other agonists with immune suppressive properties, like kynurenines. In line with this, according to a recent study, L-kynurenine induces NK cell loss in the gastric cancer microenvironment (Cui et al., 2023). At the same time, whether urolithin A itself activates any signaling pathways associated with AhR is the subject of future research.

Taken together, our data suggest that AhR may be involved in the enhancement of NK activity of PBMCs by urolithin A, but it does not appear to be the only mechanism. It is possible that urolithin A has some partial agonistic activity for the AhR, which can be considered antagonistic if other AhR ligands are present in the microenvironment. As we have shown using the multiplex cytokine assay, urolithin A decrease the production of various immune regulatory factors by PBMCs (such as fractalkine, IL-8, MCP-3) which are involved in chronic inflammation. Of note, IL-8 was shown to impair the function of NK cells via the STAT3 pathway (Wu et al., 2019).

Another way of thinking about urolithin A and AhR is that urolithin A might be the inverse agonist of AhR. Within the inverse agonist framework, receptors can be active in the absence of an activating ligand and thus display constitutive activity. As a result, some ligands can reduce the constitutive activity of a receptor. These ligands might produce the opposite effect of an agonist and are called inverse agonists (Berg and Clarke, 2018). AhR appears to be constitutively active in various pathological states (Wang et al., 2017), therefore, urolithin A might serve the role of an inverse agonist, preventing the constitutive activity of AhR.

This study has several limitations. We used only one cell type, K562, which is common for assessing PBMC cytotoxicity (Tremblay-McLean et al., 2019). Future studies should include other target cell types. Additionally, while we measured NK activity in peripheral blood, assessing PBMCs NK activity within the tumor microenvironment would be more relevant. However, *in vitro* modeling of the tumor microenvironment is challenging. Studying biopsy material from patients on urolithin A treatment would be more pertinent and is a subject for future research. In addition, the control group is small due to the difficulty in finding age-matched healthy subjects comparable to the prostate cancer patient group. The total number of participants (20 patients and 5 controls) was also relatively small, which generally increases the probability of type 1 and type 2 errors in statistical analyses; therefore, further large sample studies are warranted. We assessed the natural cytotoxic activity using a whole culture of PBMCs without studying subpopulations. While this might be a limitation, it is also more physiologically relevant as it reflects the interactions among different cell types. It is important to note that NK cells are not the sole contributors to PBMC cytotoxicity. Research indicates that CD3^+^CD56^−^ T-cells and CD19^+^ B-cells also mediate innate cytotoxicity (Sturlan et al., 2009). Furthermore, B-cells can mediate anti-cancer cytotoxicity, a mechanism often depleted in cancer patients (Janjic et al., 2022).

## 5 Conclusion

Urolithin A increases the NK activity of PBMCs in prostate cancer patients and healthy subjects. Mechanisms of this effect may include AhR modulation by urolithin A. The favorable safety profile makes possible the long-term administration of urolithin A. In this regard, clinical studies of long-term therapy with urolithin A for both prevention and add-on therapy of cancer are promising. Also, given the importance of the natural cytotoxicity of PBMCs for immune surveillance of cancer, combining various substances that enhance natural cytotoxicity is promising. For example, it is promising to study a combination of urolithin A with TLR ligands (e.g., TLR7/8), which also enhance NK activity (Khanna et al., 2021).

## Data Availability

The raw data supporting the conclusions of this article will be made available by the authors, without undue reservation.
